# Selections

**Published:** 1882-03

**Authors:** 


### Selections.

---

ON SEASICKNESS. By JAMES REGINALD STOCKER, M.B. Lond, M.R.C.P., Medical Officer, Cunard Line.

"Our remedies oft in ourselves do lie."
"*All's Well That Ends Well.*"

In drawing the attention of the profession, and it may be of the public, to this subject, I may state that I have been somewhat perplexed as to the most suitable and convenient means of doing so. The matter, in whatever way it may be treated, whether in a professional, a scientific, or even a popular way, is one of such common interest, people are so bewildered and distracted by the variety of remedies offered to their notice, and so much has been written about it in the daily and weekly journals on both sides of the Atlantic, that I might have been excused had I sought for the publication of my views in some popular journal or magazine. It is, at the same time, of whatever general interest and importance it may be to the public, one the nature of which must eventually be submitted to and determined by a scientific tribunal, and it is this that induces me to bring my own particular views forward at the present time. Some question also has arisen in my own mind as to whether the quotations I have made were quite as appropriate in a professional as in a popular publication. I trust, however, in whatever sense they may be read, they may prove instructive.

But, before proceeding to make any remarks upon the subject myself, I propose briefly to refer to certain statements that have been made by others. Some ten years ago, Professor Barker, of New York, wrote an able pamphlet on seasickness, which is practically an enlargement of a paper published by him in the New York *Medical Journal*, November, 1868. In this pamphlet he says: "The belief is very general, both in and out of the profession, that the medical art is powerless for the mitigation, relief, or cure of this malady. It is true that there are no specific drugs which will cure or even prevent seasickness, but I believe that every physician ought to be competent to give such good, sensible advice as will greatly contribute to diminish the tendency to this malady, and to mitigate and relieve the suffering and evil resulting from it." He then recounts the opportunities he has had of studying the malady, and adds that he is not sure it "can be called a disease in the proper sense of the word, for the phenomena constituting seasickness are purely physical." What he means by this is not quite clear, unless for "physical" we read "functional." Further on he discusses shortly his own views of the cause of seasickness, and remarks that "no sensible physician would therefore expect to cure seasickness by medication addressed to the stomach, or even by drugs which are supposed to act directly on the brain and its functions. The horizontal position, which to a certain degree modifies this disturbance of function, is the only approximation to a cure." In cases of prolonged seasickness, constant nausea, great nervous depression and sleeplessness, he recommends the use of potassic bromide in scruple doses, three times a day. Dr. Barker has, in my opinion, written a useful little book, for one reason, if for no other, viz., that he has vaunted no particular remedy.

Sometime after the publication of Dr. Barker's views of seasickness, M. Giraldès wrote a letter to the *Times*, lauding the use of chloral hydrate as a preventive of seasickness, which attracted considerable attention at the time, and has found a popular exponent of late in the shape of a patent or proprietary medicine. This letter was published also in the French medical periodicals at the time, the *Union Médicale* and the *Journal de Thérapeutique*, November, 1874. Twenty grains of chloral were taken before going on board, and were to be repeated during the passage (the English Channel) if necessary.

A more recent writer, Dr. Wilson, of Sandown, Isle of Wight, in a book published only last year—"The Ocean as a Health Resort," London, 1880—condemns the whole *posse comitatus* of drugs as useless. He says: "Ice bags to the spine, chloroform, chloral hydrate, prussic acid, and all the numberless remedies that have been proposed, have been weighed in the balance of experience and found wanting. Some of them," he admits, "may relieve for a time, but they interfere with the natural course of the complaint, and do more harm than good in the long run." A few lines further on he says: "When the stomach is much exhausted by sickness, small quantities of stimulants, especially in an effervescing form, will be found of great service. The best stimulants to take are brandy in small quantities, well diluted with soda water or dry champagne, either alone or mixed with soda water. When ice can be obtained, it may with very great advantage be either taken with the fluids, or allowed to dissolve in the mouth in small quantities at a time."

The Rev. Dr. Cuyler, on the other hand, in a letter published in the New York *Evangelist*, April 21, 1881, says: "Thanks to a free use every morning of Saratoga water and a careful diet, I have not been seasick. The traditional nonsense about warding off this dreaded malady by a liberal use of champagne or toddy ought to be exploded. In this case, too, 'wine is a mocker, and whoso is deceived thereby is not wise.' A good aperient, light digestible food, and fresh air are worth more than all the alcoholic potations ever concocted."

But the great modern exponent of seasickness is an American physician practicing in New York. His experience truly is great, for he appears to have been a great sufferer himself. It is curious to notice, in passing, how Dr. Beard claims to be an authority because he *is* seasick, and Dr. Barker because he *is not*. It is also interesting to observe how a system of treatment which seems almost worthy to be considered as belonging, *par excellence*, to the old school, has originated in a country which may fairly be called the "home of the homœopaths," and has been eagerly adopted, and, sometimes to their sorrow, put into practice by the public. His points are briefly these: That bromide of sodium is a specific for seasickness, that it must be used before the commencement of the voyage, and that it must be taken largely, indeed, excessively. I have examined his treatise in a spirit of careful and candid inquiry, and I must say I cannot but condemn the practice. I have no hesitation in saying that, although Dr.

Beard has been consistent and emphatic enough throughout his book in advocating the use of excessive doses of the bromide, and the necessity of commencing the treatment before going on board ship, he has, not *un ostrich-like*, most effectually and conclusively contradicted himself by a note he has innocently and ingenuously, and apparently for the purpose of confirming his theory, added to his work. This note consists principally of a letter from Dr. Hutchinson, of Providence, R. I., relating to a "combination which proved so remarkably successful. Two gentlemen, one lady, and one child were terrible sufferers with seasickness—the lady being at times delirious from functional cerebral excitement. Into an ordinary tumbler, half full of water, I put ten grains of bromide of sodium, with one-tenth grain of powdered ipecac, sat down by the bedside, and gave a teaspoonful every ten minutes. Inside of an hour all vomiting and nausea had disappeared, and the lady was asleep quietly. When she awoke, although the sea was the same as before, the sickness did not return, and she finished the voyage to Santiago de Cuba pleasantly. The same effect followed in the other cases, and as we were out almost two months, cruising around the island, I had an excellent opportunity to test this remedy. In no one instance did it fail. . . . With, I presume, more experience at sea than usually falls to the lot of physicians, this combination is the first that has proved of the smallest benefit in this contemptible disease."

I would call attention, in the first place, to the grammatical construction of this last sentence, and, in the second, to the complete refutation the letter gives to Dr. Beard's statements. It is needless to descant any further on these last two plans of treatment, except to say that the contrast between them is simply ludicrous. It is chiefly with the object of exposing the inconsistency of these and other like statements, and of calling public attention to the fact, that I venture to quote them. In addition to the popular and elegant preparations introduced of late for the relief or cure of seasickness, we may soon expect to see another, culled from the fertile brains of Dr. Beard and Dr. Hutchinson combined, and called by some such fanciful name as the proprietor may delight in and consider attractive, which may perhaps be found exceedingly useful by those who possess those two invaluable assistants to the *vis medicatrix*, ignorance and faith.

I shall now proceed to relate my own views about seasickness, and would ask my readers to remember that hitherto I have been speaking of the treatment, about which I intend to make only a few remarks, based upon the results of my own experience and that of competent and trustworthy observers.

There are some diseases, the specific fevers for example, for which there is no cure, nor is it in the course of nature that there should be. But the system, having once encountered them, is, generally speaking, no longer liable to their attacks. The only security against such diseases is either to keep well out of their way, or, by submitting to them, to undergo what may be called a personal experience of them. There are also, it cannot be denied, certain conditions of the body which render it more or less susceptible to the influence of the poisons that engender them. They depend upon age, six, condition, occupation, temperament, habits, and the like. These conditions, when favorable, appear rather to establish a kind of tolerance than to confer complete immunity. When we look around us, we cannot fail to perceive that some individuals appear at times to possess this property; their systems are either acclimatized or accustomed, or from other causes are not susceptible, to them; at any rate, they are free.

We observe a similar kind of tolerance sometimes, indeed generally, acquired by the system with regard to the special senses, particularly in certain occupations. So it is with regard to seasickness. Can we wonder that in this case, too, the system should sometimes become inured or accustomed to the sensations that produce it, and no longer amenable to their influence? Can we be surprised that in certain cases, or at certain times, men should seem to be endowed with the faculty of accommodating themselves to the singular sensations that occur on board ship?

Having said so much by way of preface to my remarks, I may now proceed to discuss the nature or the pathology of seasickness. The pneumogastric nerve is always an interesting nerve to study; in the present inquiry it is an important one: it is the key to the explanation of seasickness! It is the principal nerve of organic life: it governs the heart, the lungs, and the stomach and intestines, as well as other more remote but yet important parts. It may seem curious, but it is nevertheless a fact, that the stomach sympathizes most with the senses—the heart and lungs with the emotions, the ideas, and the intellect. If a powerful stimulus acts upon the mind, it affects principally the heart and lungs; if upon the senses, the stomach is affected. The same may be said of sedatives or soothing influences.

The pneumogastric nerve sympathizes, then, with the senses and the intellect, and plays an active part in that expression of disgust which results in vomiting. We see this thing occur as a consequence of an unpleasant impression upon any one of the special senses—the sense of taste, of smell, sight, hearing, and touch. Stimulation of the nerve, in moderation, favors digestion and the various other processes of organic life; in excess it irritates them. It directly occasions nausea, dyspepsia, flatulence, vomiting, etc.; and, indirectly, all the other sad effects of sea-sickness. The nervous centers, excited by the sensory impressions, become at last so irritable that the introduction of anything into the stomach is resented, and vomiting occurs; until sooner or later the nervous system is dominated by that potent influence for good or evil, the force of habit, and the body finally becomes accustomed to the new sensation.

I have spoken of a difference in the degree of stimulus: quantity. I will now allude to a difference in the kind: quality. There are some sensations that are pleasant and agreeable; others that are painful and disagreeable. It is not by any means always the case that the same sensations produce the same effect—*i.e.*, of pleasure or of pain—either in the same or in different persons —"what is one man's food is another man's poison." In other words, tastes differ; not only in different persons, but also at different times. An odor, *e.g.*, as of cooking, may be grateful at one time, ungrateful at another; a sound agreeable at one time, at another disagreeable. It is peculiarly so in regard to movement. One is sometimes pleasantly affected by a motion which at other times may be quite the reverse, and it always has a different effect upon some people from what it has upon others. I once met a man who said he had crossed the Atlantic eight times as a bachelor without being seasick; the ninth time he was married and had his wife with him; he was sick the whole way.

The consideration of the phenomena of seasickness has convinced me that the fifth sense, commonly called the "sense of touch," or "common sensation," is a compound sense. By its means we are able to recognize not only touch and its varieties, but also distance, form, size, weight, consistence, relation, and time, and sometimes even color and sound. "The capacity, therefore, of the hand," says Sir Charles Bell, "to ascertain the distance, size, weight, form, hardness or softness, roughness or smoothness, of objects, results from its having a compound function—from the sensibility of the proper organ of touch being combined with the consciousness of the motion of the arm, hand, and fingers."—("Bell on the Hand," 8th ed., London, 1877, page 156.)

Nor is feeling by any means the only sense that is compound in its character, for all the other senses are more or less of a complex nature. Bell, indeed, may have been mistaken in his views as to the "muscular sense" being a sense apart and distinct from the others, for it doubtless properly belongs to the various special senses with which the muscles concerned are connected, the muscular apparatus of the trunk and limbs enabling and assisting them to fulfill their functions just as much as the muscular appendages of the eye, the ear, the nose, and the tongue minister to them.

This, then, is one of the lessons that the study of seasickness teaches us. It teaches us an important physiological fact, that there is in us a sense which, without some such experience, we might perhaps be slow to recognize—the sense of passive motion. It may not indeed be so exalted a sense as others, nor so important, but it is certainly one which in seasickness deserves consideration. And after all it has its pleasant as well as its painful side, when used in moderation; it is the placid sensation that often lulls the child to sleep; it is that of the rocking-horse, the rocking-chair, horse exercise, vehicular motion of all kinds, passive movement of the body in all its forms and phases, only unpleasant, only disagreeable when used inopportunely or in excess.

There is another passage in Bell's work upon the hand (p. 148)

that bears directly upon the matter we have in hand, though from another point of view; it is this: "The nurse will tell us that the infant lies composed in her arms while she carries it up stairs, but that it is agitated when she carries it down. If an infant be laid upon the arms and dangled up and down, its body and limbs will be at rest as it is raised, but, in descending, it will struggle and make efforts. Here is the indication of a sense, an innate feeling, of danger."

Considered in the light of later years, we may perhaps trace in this observation of Bell's an instance of one of the conditions of seasickness. The study of seasickness, as has been suggested, is a complicated study, and not so simple as some appear to think. Much light has been thrown upon the subject at various times and by different writers. Barker refers it to the "sudden and recurring changes of the relation of the fluids to the solids of the body, and the nervous disturbances which result from these changes. . . . The blood by its fluidity yields more readily to the influence of descent and less easily than the solids to the ascending impulse." The *Lancet* in a leading article (Oct. 23, 1875) referred to Wollaston's theory of seasickness, which attributed the symptoms to "cerebral disturbance induced by the repeated mechanical congestion of the brain, which the repeated downward movement of the ship induces;" and showed how inefficient it was to meet the case. Ellis, of Bristol, in the same number, throws out a useful hint as to the necessity of learning to accustom one's self to the movements of the ship. Wilks and Marshall have pointed out the difference of the "pitching and tossing" or "forward" movement of a vessel from the "rolling" or "lateral" movement, and how much more exaggerated it is in one case than in the other, and how much more are its effects. Conditions which these and other observers refer to the falling weight of the viscera, I am inclined to attribute to a cause a little more remote, but produced partly by that agency. I believe that the feeling of nausea, etc., which ensues upon the falling of the vessel is the same as that due to the backward movement of the swing, or of any vehicle, the downward movement of an elevator, vertical or oblique, as well as in the dance and in the infant just referred to; and is brought about by the formation of a partial vacuum in the lung. To this cause I also attribute the condition known as *mal des montagnes*, which has been well described by M. Lortet, of Lyons ("Comptes Rendus de la Soc. des Sci.," 1869, 2, p. 707), who, by the by, ascribes it to the cooling of the body and the vitiation of the blood with carbonic acid. Dr. Macpherson (*British and Foreign Med.-Chir. Review*, 1876, p. 2. 279) gives a good account of this and other observations on the same condition. He sums up the symptoms thus: Quickened breathing and pulse, and bleeding at the nose, etc., "violent headache, sleeplessness, loss of sense and of memory, mental depression, thirst, nausea, vomiting, loss of power in the limbs. All these symptoms," he says, "remit on rest, but return on renewed exertion. All are not affected alike. . . . The amount of suffering varies in the same person at different times. . . . Those who make the most exertion suffer most. Those who are on horseback suffer less than those on foot. The lower animals are affected as much as man."

We have in seasickness conditions almost precisely similar to these, with regard to the effect as well as to the cause; a certain rarefaction of the air within the chest; a partial vacuum produced, not indeed by the rarefaction of atmosphere itself, but by the subsidence of the abdominal viscera when the vessel falls, and therefore felt more in the upright than in the horizontal position; and the continued movement of the body.

The first, *i. e.*, the want of air, is the cause more particularly of that feeling of "goneness" we so often hear complained of. I have been in the habit of recommending my patients to take a deep breath whenever they feel that sinking at the pit of the stomach, having found it by experience to be an effectual, though not infallible, means of allaying the sensation, and to this I would refer the good effect of singing or of any rhythmic movements that may tend to relieve the mind or to regulate the breathing, as well as the advantage sometimes derived from weight or pressure applied to the stomach by elastic and other belts, or bandages. Only the other day a gentleman told me he had experienced considerable relief by facing the wind and bowing forward when the vessel pitched; so many and so various are the methods men resort to in order to accommodate themselves to the change, as I

think, of the rarity of the air within the chest. The second, *i. e.*, the movement, is the cause more especially of the irritable condition of the nervous system.

The secret of the one is its direct effect upon the pulmonic branches of the pneumogastric nerve, probably the result of partial paralysis. We know that division of the pneumogastric causes vomiting; the nerve is said to exert an inhibitory effect upon the heart; it has the same effect upon the stomach. "The sensation of pain, oppression, irritation of the air passages, want of air, hunger, thirst, and satiety are dependent on this nerve. It has a regulating influence over the functions of deglutition, digestion, circulation, and respiration" (Marshall). It may, however, be due to stimulation of the pneumogastric, for the diminished resistance of the air, according to Liebig, leads to more active elastic contraction of the lung. The secret of the other is its indirect effect upon the same nerve, through the media of the nerves of feeling or common sensation, sometimes indeed through the agency of other senses, as, *e. g.*, by the sight of undulating movements, and by other unpleasant sensations. In each case, practically, the cause is of an eccentric or peripheral character.

I have said that the study of seasickness is complicated; I may now say that I recognize these two factors in its production, viz.: Irritation of the nervous centers by the ceaseless movement and other disagreeable sensations; and the sickening sensation of "want of air." I did think that my views as to the existence of the sense of passive movement being demonstrated in the case of seasickness were somewhat original; I find them expressed, however, some fifty years ago, by Herbert Mayo; and essentially confirmed by physiological writers at the present day. They tell us that seasickness is a sensori-motor act (Carpenter, Marshall), and speak of common sensation as complex, consisting of the sense of touch, temperature, the muscular sense, hunger, thirst, satiety, want of breath, fatigue, exhaustion (Marshall); but it was Mayo who was the first to recognize the fact. The index of his original observations that he gives in the preface to his work on physiology contains the following passage: "Remarks upon impressions of direction and motion; of equilibrium; hypothesis maintained of the dependence of seasickness upon the sense of disturbed equilibrium." He explains it thus : "There is a feeling attending the sudden beginning or retardation or acceleration of motion, which is independent of the sense of contact excited on one or other of the aspects of the body, and of muscular effort. When one is in a state of equable motion, as when one lies upon the ground (there being then the earth's motion only to be taken into account), or in the perfectly smooth and uniform motion of a boat gliding down a river, we *feel* that we are at rest. Any sudden alteration of the quantity of motion in us we *feel* by the sense of motion. The existence of the same sense may be rendered evident in another manner. After traveling a short distance in a rough carriage (to the city, for instance, in an omnibus), when the carriage has stopped, and you have remained on your seat for a few seconds, if you rise suddenly, you feel, for an instant on two, as if the carriage were still in motion ; this is the sense of motion *continuing after the external cause has ceased*, as the sensation produced by a flash of lightning remains in the eye longer than the light itself. It is not easy," he adds, "to say where this sense exists, whether in the muscles only, or in the joints and sinews and integuments, or in the whole frame together."

Another passage in the same work is well worth quoting here : "Nothing appears simpler or easier or more natural than maintaining our equilibrium in standing, walking, running, under common circumstances. Nor does anything appear more natural and easy than the rapid movements of the fingers of an accomplished musician playing on the piano. Both, however, are equally the results of long and at first difficult practice in the use of muscles under the guidance of more than one class of sensation. In maintaining our equilibrium we are principally assisted by the muscular sense. How much the maintenance of our equilibrium is the result of practiced sensation and muscular effort is shown by what happens on going to sea. It is long before the passenger acquires his sea legs. At first, as the ship moves, he can hardly keep his feet; the shifting lines of the vessel and surface of the water unsettle his visual stability ; the different incli nation of the planks he stands on, his muscular sense. In a short time he learns to disregard the shifting images and changing motions. or acquires facility in adopting himself (like one on horseback) to the different alteration of the line of direction in his frame."

Taking all these things into consideration; the exhausted condition of the air, the continual movement of the body, the concussion and congestion to which various organs in the body are subjected, the shaking up of the contents of the stomach, and all the unusual, unpleasant impressions that assail our other organs of special sense—the eye, the ear, the nose, and even the tongue and palate—and knowing that sickness sometimes results from any one of them, we may find it difficult rather to account for its occasional absence than to understand its frequent occurrence.

With regard to the symptoms I shall say nothing. They are tolerably familiar to most of us, and to relate them now would be to little purpose. As to the treatment, I think it lies in a nutshell. As I have said already, I propose merely to make a few general remarks upon it; not to discuss it at any length. If the views I have stated above are correct, the conclusions to be drawn therefrom are simple and obvious. Sedatives, both nervine and also stomachic (for they too will influence the terminal branches of the pneumogastric nerve), anodynes and anæsthetics, among which I include, of course, amyl nitrite, are good. So also are stimulants. Aperients are exceedingly useful. It is necessary, however, in saying this, to state that there are in this, as well as in most other complaints, certain stages or periods: the first, one of great nervous depression; the second, one of gastric and nervous irritability; the third, one of exhaustion. Things which are useful or harmless in one may be useless, and even hurtful, in another.

There are drugs which serve to diminish and even to deaden sensibility—some which serve to fortify and to strengthen it. Such drugs are no doubt of use, of much use, for the relief and prevention of seasickness; but that they are specifics I venture most directly to deny. A person's consciousness or his intellect may be so completely fuddled that he may not know what he is about; but to call that a cure is not, I venture to assert, calling things by their right names. The use of the bromides for this purpose is increasing daily, and I must say that I can not but condemn the practice of using them so indiscriminately and in such large quantities as has lately been recommended. In the *Medical Record* for July 2, 1881, is a report of a meeting of the American Neurological Association. Dr. W. A. Hammond, of New York, is reported to have said that "he had seen many cases in which acute mania had been produced with bromides, and he believed that there was a stage beyond which bromides could not be continued without producing mental aberration. The cumulative properties of the bromides should be studied more carefully than they had been. He had had four patients die while under the extensive use of bromides under his direction."

But, while I deny that there is any specific remedy or panacea for the disorder, I hasten to acknowledge that many of the conditions may be relieved by medicine. Seasickness, after all, is but a form of passive indigestion, the result of a functional neurosis in which the pneumogastric nerve is either excited or depressed. Like many other functional disorders, if anything is to be done at all for it in the way of medicine, it requires to be *treated.* One is sometimes surprised at the complete failure in some cases of a remedy which in others has proved of great service; and conversely, one is sometimes charmed with the effect of a remedy on some which has failed completely with others. With regard, for instance, to the use of alkalies as stomachic sedatives, one seems sometimes to hit upon by chance, to distinguish intuitively, or rather, perhaps, to learn by experience, what particular drug to use in each individual case. The same may be said with regard to aperients, etc. Thus would I explain the differences and agreements in the opinions of writers who have given the results of their experience to the public.

One cure, indeed, there is, viz.: custom or habit. In the course of time it almost invariably asserts itself, and "use becomes a second nature." The sooner one can accustom or habituate one's self to the altered conditions of things, the sooner will one become a good sailor. The best means of doing so is to forget it, to banish it from one's memory by the substitution of gymnastic and other exercises, and by learning the art of balancing one's self. The more one is able to forget one's self, the more one's attention can be distracted from one's own condition and diverted to other things and other people, the less will one feel the disagreeable sensation

What people want on board ship is resolution, and when the will is not sufficient and moral means have failed, the most effectual, though not by any means the most practicable, is to have recourse to force.

Captain Marryat was of this opinion, as the following passage from "Peter Simple" will conclusively show: "I admired the scenery of the Isle of Wight, looked with admiration at Alum Bay, was astonished at the Needle Rocks, and then felt so very ill that I went down below. What occurred for the next six days I can not tell. I thought that I should die every moment, and lay in my hammock or on the chest for the whole of that time, incapable of eating, drinking, or walking about. O'Brien came to me on the seventh morning, and said that if I did not exert myself I never should get well, that he was very fond of me, and had taken me under his protection, and to prove his regard, he would do for me what he would not take the trouble to do for any other youngster in the ship, which was to give me a good basting, which was a sovereign remedy for seasickness. He suited the action to the word, and drubbed me on the ribs without mercy, until I thought the breath was out of my body, and then he took a rope's end and thrashed me until I obeyed his orders to go on deck immediately. Before he came to me I could never have believed it possible that I could have obeyed him; but somehow or another I did contrive to crawl up the ladder to the main deck, where I sat down on the shot racks and cried bitterly. What would I have given to have been at home again! It was not my fault that I was the greatest fool in the family, yet how was I punished for it! If this was kindness from O'Brien, what had I to expect from those who were not partial to me? But, by degrees, I recovered myself, and certainly felt a great deal better, and that night I slept very soundly. The next morning O'Brien came to me again. 'It's a nasty slow fever, that seasickness, my Peter, and we must drive it out of you;' and then he commenced a repetition of yesterday's remedy until I was almost a jelly. Whether the fear of being thrashed drove away my seasickness, or whatever might be the real cause of it, I do not know, but this is certain, that I felt no more of it after the second beating, and the next morning when I awoke I was very hungry."

I may add that this is a mode of treatment perfectly well known in the merchant service, and found to be of inestimable value even at the very commencement of the complaint.—*New York Medical Journal.*

---

IN WHAT CASES SHALL THE MEDICAL EXAMINER DECLINE TO VIEW A DEAD BODY? By Medical Examiner ALFRED HOSMER, M.D., of Watertown.*

The medical examiner forms a conspicuous and important element in that vast system of protection which it has been found necessary to establish for the community. In one of his relations to the public he is, but in no menial sense, a servant, and as such he should be willing, prompt and obedient. With this idea in mind I have never failed to report for duty whenever a requisition has been made for my official services. Yet it has sometimes happened that after reaching the place where immediate action on my part was expected, I have been forced to the conclusion that the case in hand not being among those contemplated and provided for by the statute of 1877, I had derived no authority from that act to take cognizance of it, and have therefore declined to hold a view in the legal sense of the phrase.

For instance, one morning a police officer requested me to give immediate attention to a case which had occurred five miles away, in the district of Medical Examiner ——, then absent. At much personal inconvenience I responded to the summons without delay. I found in the custody of the undertaker the body of an elderly man, who, early in the day, while in company with a friend, had died suddenly in the street. Careful interrogation failed to discover any suspicion that the death was not a natural one, and also elicited the statement that the deceased had recently consulted a neighboring physician. The fact that the death had been both public and sudden was assigned as the only reason for calling a medical examiner. Yet a little reflection will satisfy all that neither of these features in that case could be considered *per se* as proof of "death by violence." Sudden deaths

*Read before the Massachusetts Medico-Legal Society.

from natural causes are unquestioned, although their frequency has not yet been exactly determined. Their origin and relations form a subject which deserves a full elucidation before the Society. Public deaths are not likely to be unnatural ones unless they occur under circumstances which plainly show their violent character. There can be no doubt as to the nature of the fatal results that follow a social disturbance, which may exist in any and all degrees, from a simple individual assault to a riot of the largest proportions. There is no difference of opinion as to the kind of death which overtakes the victims of a disaster or awaits those who lose their lives through that agency which, with a careless freedom of language, we are accustomed to speak of as accident.

To return to the point whence this digression started, I sought the physician who had attended the deceased. On the demand of the instant he could not furnish the information I desired. He promised to investigate and report; in a very short time he wrote to me as follows: "Mr. —— had a marked stenosis of the mitral valve; was moderately comfortable with care. I learn that he had been drinking hard a day or two preceding his death." Such evidence as this fully confirmed me as to the correctness of the conclusion at which I had arrived, and upon which I had acted, when, in the presence of the dead body, I had expressed the opinion that the case did not come within the province of the medical examiner. There was no duty prescribed by law that I could perform, there was no service that I was qualified to render, and, rendering none, I considered myself entitled to no compensation, and have never claimed any.

An answer to the question which forms our subject must be obtained from a careful study of the law, of which the seventh section is as follows: "Medical examiners shall make examinations as hereinafter provided upon the view of such *persons* only* as are *supposed* to have come to their death by *violence*."

What is a death from violence within the meaning of the statute? In a general statement it is one with the causation of which human agency is connected directly or indirectly; through default of a reasonable and necessary precaution; through neg-

*The *italics* form no part of the original law.

lect to secure protection against danger known to exist or to be incurred; through force, mental, mechanical, medicinal, chemical or toxic, exercised and applied either ignorantly and unconsciously, or under the impulse of excitement, without premeditation or with deliberation and improper purpose. The definition requires some explanation and limitation, and will be better understood if we constantly bear in mind the fact that the high function of the medical examiner is to assist in the detection of *crime against life.* But the location of the line which separates guilt from innocence is fixed by the provisions of statute law. Violence of death has no constant relation to legal prohibition and penalty, and is not confined to the unlawful destruction of life. In deference to the necessity and the right of self-defense the shooting of an unprovoked assailant may receive no sentence of condemnation. Yet such a death must primarily be treated as a violent one, for the true character of the deed can be determined by judicial inquiry alone, and exoneration is attained only with the verdict which concludes the inquest. On the other hand, death by violence cannot be held to include such human agency as that, for instance, which in the form of parental indiscretion has contributed so freely to the mortality of infants. Neither can it mean those deaths which, also traced to a similar origin through what some one might have done but has failed to do in a preventive sense, are often sudden, always premature, accidental as we call them. The child who, for want of attention, is allowed to be drowned in a cistern or scalded to death in a vessel of hot water; the boy who in his carelessness falls from a tree and breaks his neck; the laborer who in perfect health and in pursuit of his daily work receives a heavy blow upon the abdomen and three days latter suddenly passes into a collapse from which he never rallies; the man of business who with hasty and ungarded steps collides with a post in the street and sustains injuries which are ultimately fatal; the victim of melancholy who wanders from home and is lost, and whose remains are found months afterwards with everything untouched except by the process of decay; those who perish by the unmistakable consequences of great natural commotions, such as thunder-storms and tornadoes; not one of these furnishes an example of death by violence in the medical examiner sense; no one of them dies by the method provided in the easy and uninterrupted order of nature; but on the other hand they do not die through an unlawful course of action on the part of any person.

"Supposed to have come to their death by violence." The word "supposed" imparts a degree of elasticity to the section, and without being intended to exclude those cases which derive a known character from obvious violence, enables it to cover a field the contents of which, as being unexplored, would otherwise be uncertain; and in which either the rights of good personal reputation or the demands of justice require that doubts should be replaced with facts. The term in question seems, at first sight, to be a vague one; it implies a mental process, but carries no reference to the conditions which shall render that process a sound and valid one. We must believe that the word is intended to mean the average opinion of intelligent minds whose conclusions, unmixed with the elements of credulity and timidity, represent the preponderance of well-considered testimony. In addition to this, violence may reasonably be "supposed" in those cases which, though not presenting *prima facie* a suspicious aspect, cannot be proved by a very large probability to fall within the range of the operation of the natural causes of death. Whenever violence is not self-evident but only supposed, it is the duty of the medical examiner, before taking any action, to assure himself that the theory of illegal force commands a plausible support. His position is not a judicial one, yet his decisions may be final. He must judge of the applicability of the law to the case in hand. He must exercise that discretion which takes precedence of learning in the enumeration of qualities which determine the selection of candidates for office. Thus will he aid in establishing those principles upon which must be based every rule of official action, and in teaching the public to understand and accept those distinctions which are of fundamental importance, and which cannot be neglected without endangering his own integrity and dignity, and doing injury to the cause of law and order.

(*Concluded in April Number.*)

NO BACTERIA IN DIPHTHERIA.—(*The Medical Record*, Vol. XXI, p. 150). Dr. R. R. Gregg calls attention to the following facts in regard to diphtheria, and, as he thinks, general misinter-ons:

First,—" There is not the slightest difference to be found recorded by the best observers, between the three classified forms of so-called bacteria in diphtheria, namely: spherical, rod-like and spiral, and the three exactly corresponding forms of coagulating fibrin, namely: granular, thread-like and spiral"

Second,—Where blood congests, there fibrin soon commences to coagulate, first into granules; then these join to form fibrils, and later spirals, unless prevented by the attachment of their ends. Lehmann, in speaking of coagulation, says: " The same process goes on in the vessels of the living organism as soon as the blood ceases to circulate." And Wood, in his " Practice of Medicine," says, when speaking of the organization of inflammatory exudates: " As it first escapes it is a homogeneous, transparent fluid, but soon afterward, if examined by a microscope, it is found to contain multitudes of fibrils " and " great numbers of minute granules of different sizes." Thus it appears, according to Lehmann, that in blood which stagnates, as it always does under congestion and inflammation, the fibrin at once begins to coagulate; and according to Wood, under the same circumstances, where observers say their bacteria are found in the greatest abundance, the granules and fibrils of fibrin are found in the greatest profusion. Therefore, " those who claim the presence of bacteria, or vegetable organisms, in the same part where the exactly corresponding forms of fibrin develop rapidly and in great numbers, must show the clearest distinction between these two sets of forms," and the relative proportion of each, or withdraw their claim.

Third,—In diphtheria fibrin is always in excess in the blood, and the false membranes, heart-clots, or thrombi and emboli are formed of it. These cannot be made without first the formation of granules and then the joining of these into threads or rods. Therefore, since these granules and threads must exist in or upon congested and inflamed surfaces, when the corresponding forms of so-called bacteria begin to develop, there is no necessity for accounting for the presence of granules, threads or rods by regarding them as vegetable growths or bacteria.

The author also criticises the statement of Prof. H. C. Wood, that "the membrane bears everywhere the appearance of little balls about the size of blood corpuscles, either singly or in groups of four or more. When the blood is examined, the white blood-cells are found to contain these micrococci, moving about to the number of forty or fifty in each cell. By experiment it was found that this infinitesimal plant fastens upon the white corpuscles, and multiplies its cells," changing the interior of the corpuscle until it is destroyed, and finally bursting it, and thus setting loose the plants in an irregular mass. "Thus increased, they poison the blood, choke the vessels, and are found in myriad numbers in the spleen and bone marrow, where the blood is manufactured."

The author claims, from a very careful study of the blood-corpuscles under all circumstances, which study has continued through a period of twenty years, that the micrococci which Prof. Wood observed in the white blood-cells are nothing more nor less than the granules which naturally constitute every such cell; and "the little balls about the size of blood-corpuscles," which were observed in the membrane were simply decolorized red blood-corpuscles, distended and made globular by the suppurative processes. Plates illustrating the granular structure of the white blood-cell can be found in "Lehmann's Physiological Chemistry" and T. B. Lippincott & Co.'s edition of "Virchow's Cellular Pathology." The claim that the micrococci or "granules seize upon and destroy blood-cells would seem to be an imaginary and gratuitous assumption." That the blood-cells burst or "melt down," and release their component granules, is true, because they are dead and undergoing disorganization. Until the granules become putrid they do not poison or destroy other tissues.

No observer has yet pointed out the distinction that exists between the granules formed from disintegrating blood-cells and the granules into which fibrin first coagulates. The author claims that the first "lead to suppurations, and possibly to septicæmia, but *never* to false membranes;" while the latter lead "rapidly and directly on to the organization of false membranes and heart-clots. 'The infinitesimal plant found in myriad numbers in the spleen' by Prof. Wood was unquestionably the granules of disintegrated blood-cells, which are sent to the spleen to be still further disintegrated, preparatory to being cast out of the system through the bowels, as refuse matter. Besides, the blood-globules are not made in the spleen, but by the mesenteric glands, and disintegrated by the spleen and liver at the close of their life, whether of old age or disease."

